# Cross-Talk between Mechanosensitive Ion Channels and Calcium Regulatory Proteins in Cardiovascular Health and Disease

**DOI:** 10.3390/ijms22168782

**Published:** 2021-08-16

**Authors:** Yaping Wang, Jian Shi, Xiaoyong Tong

**Affiliations:** 1 Chongqing Key Laboratory of Natural Product Synthesis and Drug Research, School of Pharmaceutical Sciences, Chongqing University, Chongqing 401331, China; W2515948466@163.com; 2 Leeds Institute of Cardiovascular and Metabolic Medicine, School of Medicine, University of Leeds, Leeds LS2 9JT, UK

**Keywords:** mechanosensitive ion channels, Piezo channels, TRP channels, Ca^2+^, NCX, Orai, STIM, IP3R, SERCA, cardiovascular disease

## Abstract

Mechanosensitive ion channels are widely expressed in the cardiovascular system. They translate mechanical forces including shear stress and stretch into biological signals. The most prominent biological signal through which the cardiovascular physiological activity is initiated or maintained are intracellular calcium ions (Ca^2+^). Growing evidence show that the Ca^2+^ entry mediated by mechanosensitive ion channels is also precisely regulated by a variety of key proteins which are distributed in the cell membrane or endoplasmic reticulum. Recent studies have revealed that mechanosensitive ion channels can even physically interact with Ca^2+^ regulatory proteins and these interactions have wide implications for physiology and pathophysiology. Therefore, this paper reviews the cross-talk between mechanosensitive ion channels and some key Ca^2+^ regulatory proteins in the maintenance of calcium homeostasis and its relevance to cardiovascular health and disease.

## 1. Introduction

Despite decades of efforts, cardiovascular disease is still the number one killer in the world. The latest data show that one in six elderly people dies of cardiovascular disease. In 2019, ischemic heart disease and stroke were reported to be the leading causes for disability in the age groups of 50–74 and 75 years or above [1]. As a dominant second messenger, Ca^2+^ plays an important role in the cardiovascular health and diseases. For example, dietary calcium supplement and its retention reduce cardiovascular response to sodium stress in Black people [2]. There are still some controversies over the cardiovascular effects of high Ca^2+^ intake in the diet, and whether the relationship between Ca^2+^ intake and cardiovascular disease risk is J- or U-shape [3]. Ca^2+^ is pivotal in maintaining the functions of endothelial cells, smooth muscle cells (SMCs) and cardiomyocytes. It controls the contraction or relaxation of arteries and heart and regulates blood pressure and cardiac functions [4,5,6,7]. 

The cell membrane controls the balance between intracellular and extracellular Ca^2+^ via various proteins, and thus maintains Ca^2+^ homeostasis. Plasma membrane Ca^2+^ ATPase (PMCA), voltage-gated calcium channel (VGCC), Na^+^/Ca^2+^ exchanger (NCX), and Orai have been identified as the main calcium regulatory proteins on the cell membrane [8,9,10,11]. Endoplasmic reticulum (ER) as an important Ca^2+^ reservoir in the cell also contains some calcium regulatory proteins. Such proteins in the ER include stromal interaction molecules (STIM), inositol 1,4,5-trisphosphate receptor (IP3R), and sarco/endoplasmic reticulum calcium-ATPase (SERCA). All these proteins are critical in controlling cell functions, such as growth, migration, apoptosis, and metabolism [12,13,14]. As a general feedback mechanism, these key Ca^2+^ regulatory proteins are also regulated by intracellular Ca^2+^ level.

Mechanical forces are crucial for cardiovascular functions and therefore the discoveries of mechanosensitive ion channels represent a major breakthrough in understanding cardiovascular mechanobiology. In particular, the endothelium in the cardiovascular system is subjected to regular mechanical stimulus evoked by blood flow. The discovered mechanosensitive ion channels including Piezo channels and transient receptor potential (TRP) channels can conduct the entry of cationic ions, particularly Ca^2+^, in response to the stimulus from shear stress of blood flow [15,16]. Both Piezo and TRP channels are closely linked to the development of cardiovascular disease. In some cardiovascular diseases, such as hypertension, atherosclerosis, or aneurysmal plaques, altered mechanical stress which can directly activate mechanosensitive ion channels has been reported [17]. 

**Figure 1:**
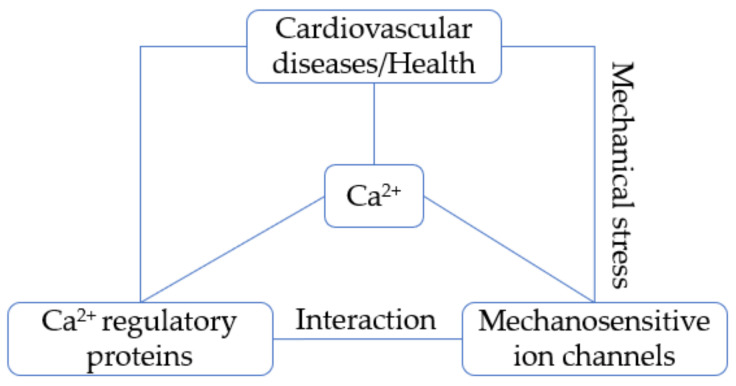
Ca^2+^ links Ca^2+^ regulatory proteins with mechanosensitive ion channels to regulate cardiovascular health and diseases. The mechanical stress generated in either cardiovascular health or disease directly stimulates mechanosensitive ion channels and induces Ca^2+^ entry. Then the Ca^2+^ regulatory proteins sensing the changes of Ca^2+^ cross-talk to or interact with mechanosensitive ion channels, which contributes to the development of cardiovascular disease or health.

Ca^2+^ regulatory proteins sensitize any subtle change of intracellular Ca^2+^. It has emerged that these proteins can cross-talk to mechanosensitive ion channels (Figure 1) and such cross-talk can even take place with direct and physical interactions between them, such as Piezo and SERCA [18]. This paper reviews the cross-talk between mechanosensitive ion channels and some key Ca^2+^ regulatory proteins in the regulation of Ca^2+^ homeostasis from the perspective of cardiovascular health and disease. In-depth understanding of the structure, function, and cross-talk of these proteins will help us to unravel the pathogenesis of cardiovascular disease and further develop novel therapeutic strategies. It is noted that the cross-talk or interaction between mechanical ion channels, PMCA, VGCC in cardiovascular system awaits further investigation and hence is not reviewed in this paper.

## 2. Ca^2+^ Regulatory Proteins in Cardiovascular System

### 2.1. NCX

NCX mainly works in the forward mode, which uses the electrochemical gradient driven by Na^+^ to expel Ca^2+^ from cells in order to maintain the concentration of Ca^2+^ required for physiological activities, and the ion stoichiometric ratio is 3Na^+^:1Ca^2+^ [19]. There are three types of NCX: NCX1, NCX2, and NCX3 [20]. Structural studies revealed that eukaryotic NCX protein consists of 10 transmembrane helixes. There is a large cytoplasmic regulatory loop between transmembrane helix 5 and 6. This loop includes two regulatory (Ca^2+^)-binding domains 1 and 2 [21,22], which adjust the rate of from cells to adapt to the dynamic Ca^2+^ oscillation [21,23,24]. The Ca^2+^ extrusion usually leads to smooth muscle relaxation, so that the vascular tension is reduced [25]. However, under some extreme conditions, such as high concentration of intracellular Na^+^ or high positive membrane potential, NCX works in a reverse mode to evoke Ca^2+^ influx instead of the typical extrusion [23], which can then lead to contraction of SMCs and artery [26,27]. Interestingly, the forward mode of NCX can be changed to its reverse mode by the increase of cytosolic Na^+^ and membrane potential [28], suggesting a feedback regulatory mechanism may exist [19].

NCX regulates many essential physiological events, such as muscle excitation-contraction or blood pressure regulation [29,30]. The deletion of NCX causes the loss of NCX function in myocardium and consequentially results in embryonic death [25]. The altered expression and regulation of NCXs could disrupt Ca^2+^ homeostasis and initiate molecular and cellular remodeling in various tissues, which is related to hypertension and heart failure. Inhibitors of NCX can improve myocardial function in patients with heart failure and bring the Ca^2+^ hyper-responsiveness back to normal in vascular SMCs from hypertensive patients [26,31]. NCX1 in smooth muscle and endothelium could play opposite roles in regulating blood pressure. Arterial blood pressure is correlated with the expression level of NCX1 in vascular SMCs [26]. The increased expression of vascular NCX1 is associated with the vasoconstriction in several animal models of salt-dependent hypertension [26]. Furthermore, reduced arterial myogenic tone and low blood pressure were observed in vascular smooth muscle NCX1 conditional knockout mice. However, for the mice with NCX1 overexpression in vascular SMCs, high blood pressure and vasoconstriction even accompanied with increased expression of transient receptor potential canonical channel (TRPC) 6 were reported [26], which suggests that NCX could control arterial constriction and regulate blood pressure by cross-talking to TRPC6 channels [26]. In the mesentery constricted by phenylephrine, antagonists of NCX reverse mode eliminate acetylcholine-evoked nitric oxide production in intact mesenteric arteries and inhibit acetylcholine- or ATP-induced increase of intracellular Ca^2+^ in cultured endothelial cells, indicating that the activation of NCX reverse mode can play an important role in mediating the acetylcholine-induced vasodilation in resistance arterial endothelial cells [32].

### 2.2. Orai

Orai is a highly (Ca^2+^)-selective ion channel in the plasma membrane formed by four transmembrane domains. Orai family includes Orai1, Orai2, and Orai3 [8]. The calcium release-activated calcium (CRAC) channels are composed of Orai and STIM, representing a typical voltage independent store-operated Ca^2+^ entry (SOCE) [33,34]. Store-operated Ca^2+^ channels fine tune Ca^2+^ entry in both cardiomyocytes and SMCs, and they are activated once the Ca^2+^ store in ER or sarcoplasmic reticulum (SR) is depleted or the level of cytosolic Ca^2+^ is lowered, thereby facilitating agonist-induced Ca^2+^ influx. It has also been suggested that STIM1, Orai and TRPC channels could form the molecular basis of SOCE in some types of cells and their intricate interactions control the entry of Ca^2+^ into cells to regulate numerous physiological processes [35]. Orai1 in plasma membrane and STIM1 in ER conduct Orai-STIM signaling at the membrane junction between ER and plasma membrane, and they are the bona fide molecular components of SOCE and CRAC [8,36]. Once the ER Ca^2+^ store is depleted, STIM1 protein can move to the plasma membrane and activate Orai and TRPC channels, allowing extracellular Ca^2+^ to enter the cytoplasm [8,36]. Orai2 and Orai3 channels have been discovered to be key players in regenerative Ca^2+^ oscillations induced by physiological receptor activation, while Orai1 is not necessarily involved in this process. However, the binding of Orai2 and Orai3 to Orai1 could expand the sensitivity range of receptor-activated Ca^2+^ signals [37]. 

Orai plays a critical role in regulating cardiovascular function in both health and disease [35,38,39]. Orai1 protein deficiency leads to heart failure in zebrafish [40]. The knockout of Orai3 in cardiomyocyte causes dilated cardiomyopathy and heart failure in mice [41]. Both Orai1 and Orai3 are the phenotype modulators of vascular SMCs. Orai1 is upregulated in SMCs during vascular injury. The downregulation of Orai1 inhibits SMC proliferation and reduces neointima formation following balloon injury of rat carotids [42]. Orai3 is also upregulated in neointimal SMCs in rat balloon injured carotid artery, and the knockdown of Orai3 inhibits neointima formation [43]. The transformation of vascular SMC phenotypes is one of the pathological characteristics in chronic hypertension, and the synergistic action between Orai and STIM mediates Ca^2+^ entry and drives the fibroproliferative gene program [44]. Orai facilitates Ca^2+^ entry and is a potential therapeutic target for the treatment of hypertension [35]. Most cardiovascular diseases are closely associated with cellular remodeling, and Ca^2+^ signaling pathways have emerged as important regulators of smooth muscle, endothelial, epithelial, platelet, and immune cell remodeling [45]. Ca^2+^-permeable Orai channel is also important for endothelial cell proliferation and angiogenesis [46,47]. In terms of vascular physiology and functional regulation, Orai1 appears to trigger the increase of vascular permeability, which is an early marker of atherogenesis. Knockdown of Orai1 reduces the histone 1-induced hyperpermeability in endothelial cells [48]. ApoE knockout mice are a common model for atherosclerosis. A high fat diet can upregulate the expression of Orai1 mRNA and protein in aortic tissue. SiRNA knockdown of Orai1 can reduce the size of atherosclerotic plaque [49]. The migration of neutrophil is another hallmark in atherosclerosis, and during this process Orai1 is required for neutrophil migration to the inflammatory endothelium [45]. All these experimental evidence show that Orai1 expression is associated with development of atherogenesis. Moreover, Orai often acts in conjunction with STIM to form CRAC, which can be responsible for many physiological functions or the development of various cardiovascular diseases [35,39,50]. 

### 2.3. STIM 

STIM is a single pass transmembrane protein residing in the ER. It contains two homologous proteins, STIM1 and STIM2 [51,52]. The function of STIM is to sense the concentration of Ca^2+^ in ER and makes appropriate response through conformational change to regulate Ca^2+^ homeostasis [39,53]. STIM1 stays at a closed state when ER lumen is filled with Ca^2+^ and transitions to an open state when Ca^2+^ in the ER lumen is decreased [54]. The Ca^2+^-sensitive domain in the STIM N-terminal senses Ca^2+^ level of ER ranging from 100 to 400 µM [52,55], and the C-terminal of STIM interacts with Orai to form CRAC channel to induce Ca^2+^ influx [52,56]. As mentioned above, the interactions between STIM1 and Orai1 regulate physiological and pathological functions [35,39,50].

STIM is involved in both the cardiac physiological functions and the cardiac disease development. STIM is essential for the maintenance of myocardial contractility, and its knockout leads to a reduction in left ventricular contractility. STIM1 is expressed more abundantly in early cardiomyocytes than in somatic cells. Cardiomyocyte-STIM1-specific knockout mice exhibit dilated cardiomyopathy and cardiac fibrosis with increased stress biomarkers and altered organelle morphology in the heart, suggesting that STIM1 can regulate myocardial development and heart function [39]. However, the spatially differential distribution of STIM1-triggered Ca^2+^ signaling generates the Ca^2+^ microdomain that regulates myofilament remodeling and activates pro-hypertrophic factors locally, and as a consequence, pathological cardiac hypertrophy is induced [57]. The STIM1-guided Ca^2+^ signaling is also involved in thrombosis. The aggregation of platelets at the site of thrombosis requires the increase of intracellular Ca^2+^ concentration. STIM1 is involved in this process through maintaining a high Ca^2+^ concentration. In addition, STIM1 stabilizes the thrombus by promoting the expression of phosphatidylserine in plasma membrane [39,58]. The upregulation of STIM induces fibroproliferative gene expression and vascular SMC remodeling, which eventually leads to chronic hypertension [44]. The cell proliferation and migration promoted by STIM1 are also involved in atherosclerosis [59,60]. Oxidized low-density lipoprotein (ox-LDL) can increase the expression of STIM1, and then promote cell proliferation and migration in mouse aortic SMCs. Silencing STIM1 inhibits ox-LDL-induced cell proliferation and migration and hence suppresses atherosclerosis [59,60]. The role of STIM1 in the pathogenesis of these diseases suggests that specific inhibition of STIM1 may contribute to the treatment of these diseases. STIM2 is another important protein for health. Studies on STIM2 deficient mice show that they gradually die from 4 to 8 weeks [61].STIM2 has similar functional effects to STIM1 in some aspects. Both proteins can promote vascular remodeling by inducing the transformation of phenotypes in pulmonary artery SMCs [62]. 

### 2.4. IP3R

IP3R is a tetrameric channel consisting of four glycoproteins in the ER or SR. So far, three types of IP3R channels have been identified: IP3R1, IP3R2, and IP3R3 [63]. IP3R has four structural regions: IP3 binding region, central regulatory region, transmembrane domain, and C-terminal region. It can be activated by the selective ligand inositol 1,4,5-triphosphate (IP3) and is permeable to Ca^2+^ [64]. All these isoforms of IP3R can be expressed in vascular SMCs. They are important for the physiological functioning of the cardiovascular system [65]. IP3R is one of the major sources for intracellular Ca^2+^ release. The overexpression of IP3R enhances ER Ca^2+^ depletion, which reduces ER intraluminal Ca^2+^ concentration in the vicinity of STIM1 and then activates Orai ion channels [66]. In response to increased IP3 or decreased Ca^2+^ in ER, IP3Rs empty Ca^2+^ stored in the ER and activate Ca^2+^ inward flow [67]. IP3R also functions on the membrane contact sites between ER and mitochondria to transport Ca^2+^ from ER to mitochondria. Each isoform of IP3R can mediate this contact and Ca^2+^ transport, but IP3R2 is the most efficient one in delivering Ca^2+^ to mitochondria from ER [68,69]. The voltage-dependent anion channel on the outer mitochondrial membrane can also enhance Ca^2+^ accumulation through physical interaction with IP3R [70], which is vital for the maintenance of mitochondrial function.

Under physiological conditions, IP3R signal controls the contraction, migration, and proliferation of vascular SMCs. However, under the pathological conditions, IP3R is involved in the development of atherosclerosis and hypertension [71]. IP3R is activated following the stimulation of G-protein coupled receptors and binds to STIM1 upon Ca^2+^ depletion in ER. The association of IP3R-STIM1 increases Ca^2+^ inward flow [66]. When IP3 binds with IP3Rs, vasoconstriction and hypertension can be induced as a consequence to the increased concentration of cytoplasmic Ca^2+^ released from the ER. The deletion of IP3Rs reduces the contractile response to vasoconstrictors and even reverses the pathological states [65]. In the heart, IP3R-mediated Ca^2+^ release ensures the integrity of cardiac excitation-contraction coupling, which forms the basis of the heartbeat [72]. The dysfunction of IP3R in cardiomyocytes leads to the disturbance of local Ca^2+^ homeostasis, which is closely related to congenital diseases, increased risk of arrhythmia, decreased contractility, or heart failure related arrhythmias [73]. The expression of IP3R is upregulated in atrial fibrillation, and inhibition of IP3R can significantly reduce the occurrence and duration of atrial fibrillation. Therefore, IP3R may emerge as a new target for the treatment of atrial fibrillation [74]. 

### 2.5. SERCA

SERCA is a Ca^2+^ transporter located on SR/ER and is mainly responsible for the transport of cytoplasmic Ca^2+^ back to SR/ER. SERCA isoform is encoded by SERCA1, SERCA2, or SERCA3 genes. Each isoform may have differential roles in different tissues or cells [75]. There are four functional domains (M, N, P, and A) and a polypeptide chain in SERCA protein. The M domain contains transmembrane components and Ca^2+^ binding sites, while N, P and A located in the sarcoplasm are responsible for ATP hydrolysis [76]. Each ATP hydrolysis can transport 2 Ca^2+^ to the ER lumen in exchange for 1 H^+^ [77]. SERCA2a is the major isoform of cardiac SERCA, while SERCA2b is the major one of vascular SERCA. 

The influx of Ca^2+^ into SR/ER mediated by SERCA2 is necessary for the relaxation of cardiomyocytes and blood vessels. The disruption of SERCA2 activity leads to ER stress and cardiovascular disease [76]. Hormones, phospholamban and sarcolipin are the common regulators of SERCA. Especially, phospholamban plays a major role in regulating SERCA, and its interaction with SERCA2a reduces the binding affinity of SERCA2a to Ca^2+^ at low cytoplasmic Ca^2+^ concentration [78]. The downregulation of SERCA2a is found in failing heart and atherosclerotic vessels [79]. The decreased protein level of SERCA2a and p16-phospholamban leads to left ventricular diastolic dysfunction and elevated arterial blood pressure [80]. Activation of SERCA can accelerate the reuptake of Ca^2+^ by SR, which would improve the diastolic dysfunction of myocardium, and result in strong antiarrhythmic effect [81,82]. Our groups found that the *S*-glutathiolation of the amino acid residue Cys674 (C674) is key to the increase of the activity in SERCA2 under physiological conditions [83,84], but this post-translational protein modification is prevented by the irreversible oxidation of C674 thiol in pathology hallmarked by high level of ROS, including atherosclerosis, aortic aneurysms, aging and hypertension [83,85,86,87]. The substitution of the SERCA2 C674 by serine causes impaired angiogenesis following hindlimb ischemia by interrupting the physiological functions of endothelial cells and macrophage [88,89], increases blood pressure by inducing sodium retention and ER stress in the kidney [87], exacerbates angiotensin II-induced aortic aneurysm by switching the phenotypes in aortic SMCs [90], aggravates high fat diet-induced aortic atherosclerosis by evoking inflammatory response in endothelial cells and macrophage (our unpublished data), promotes pulmonary vascular remodeling, and protects against left ventricular dilation caused by chronic ascending aortic constriction [91]. All these data imply that the redox state of C674 and the function of SERCA2 are critical to the maintenance of cardiovascular homeostasis. 

Currently, there are some ongoing clinical trials for the drugs specifically targeting these Ca^2+^ regulatory proteins in the cardiovascular system, as shown in Table 1. These trials provide promising opportunities for the treatment of cardiovascular diseases.

## 3. Mechanosensitive Ion Channels in Cardiovascular System

### 3.1. Piezo Channels

Piezo channels are cation channels activated by mechanical force. They have two subtypes: Piezo1 and Piezo2. Piezo1 is the major one distributed in cardiovascular system. Upon mechanical stimulus, Piezo channels convert the mechanical signals into biological signals to participate in cellular physiological events. When Piezo1 channels sense shear stress in cardiovascular system, they conduct the inward flow of cationic ions, especially Ca^2+^ [99]. Piezo1 in the plasma membrane of endothelial cells can cause depolarization in response to the blood flow [17,100]. Since the discovery of Piezo channels in 2010, more and more evidence have revealed its importance in physiological activities and disease development [99,101,102]. Cryo-electron microscopy studies show that mouse Piezo1 is a three-bladed, propeller-shaped homologous trimeric protein consisting of a central cap, three peripheral blade-like structures on the outside of the cell, three long beams on the inside of the cell connecting the blades to the cap, and a transmembrane region between these features [103,104,105,106]. 

The systemic knockout or specific endothelial disruption of Piezo1 in mice severely disrupts angiogenesis and results in embryonic death within days of cardiac pulsation [107]. Endothelial Piezo1 mediates atheroprotective or atheroprone signaling depending on the flow pattern [108]. Disturbed flow leads to Piezo1-mediated inflammation, and Piezo1 deficiency in endothelium reduces atherosclerosis [108]. Piezo1 can also regulate vascular tone and arterial blood pressure through increasing intracellular cationic ions and modulating AKT and eNOS phosphorylation [109]. Especially, Piezo1 has an interesting dichotomy in endothelial cells [100,101]. When the channel opens, it causes intracellular elevation Ca^2+^ and Na^+^. The latter can depolarize endothelial cells. In this case, depolarization in the endothelium can be transduced to the vascular SMCs in which voltage-gated Ca^2+^ channels could be activated to drive vasoconstriction [110]. Such vasoconstriction is beneficial in increasing physical performance by re-distributing blood supply in the body to support sustainability of physical activities [110]. The important roles Piezo1 play in the health and disease indicate that the channel would be potentially key therapeutic target for the treatment of some cardiovascular diseases.

### 3.2. TRP Channels

Based on amino acid sequence, TRP channels can be classified into seven subfamilies: TRPC, TRPV, TRPM, TRPA, TRPP, TRPML, and TRPN. They share some common structural features including six transmembrane domains and a pore lining between the fifth and sixth transmembrane domains [111,112]. Most TRP channels are Ca^2+^-permeable, but their Ca^2+^ permeability varies [113]. For example, the permeability of TRPV4 to Ca^2+^ is much greater than that of TRPC6 [114], while TRPM4 channel is almost impermeable to Ca^2+^ [115]. As mechanosensitive ion channels, TRP proteins can regulate contraction, relaxation, proliferation, differentiation, and apoptosis in cardiovascular physiology or pathophysiology [116]. Meanwhile, they can also be stimulated by the depletion of SR/ER Ca^2+^ stores [113,117]. Recent studies have shown that TRPC1 channels can form store-operated Ca^2+^ channels with a contractile phenotype independently of Orai1 in vascular SMCs [118]. 

TRP channels control acute hemodynamics and regulate cardiac remodeling through the modulation of Ca^2+^ signaling, which is relevant to physiological and pathological functions in the heart [119]. Shear stress is almost universally associated with vasodilation in the peripheral vasculature, and there is considerable evidence that TRPV4 channel-mediated Ca^2+^ entry following stimulation of shear stress mediates the vasodilatory response in endothelial cells [120,121]. TRPV2 channel is mainly expressed in the sarcomeres and ER to maintain appropriate mechano-electric coupling in cardiomyocyte. Under pathological conditions, TRPV2 is translocated to the sarcolemma to mediate an abnormal Ca^2+^ entry [122]. TRPA1 channel activated by pressure overload increases Ca^2+^ inward flow and activates Ca^2+^-dependent pathways, which would lead to diastolic-mediated heart failure in cardiomyocytes [123,124]. TRPA1 expression is increased in failing hearts. Inhibition of TRPA1 channel improves myocardial hypertrophy and heart failure [125]. TRPA1 channels in cardiomyocytes contribute nearly 40% of the increase of intracellular Ca^2+^ concentration under physiological conditions, which then induces nitric oxide release and vasodilation [124,126]. TRPA1 has a similar role to TRPV4 in lowering blood pressure through such vasodilation [127,128]. From these perspectives, there are some similarities between TRP channels and the above-mentioned Piezo channels in regulating blood pressure. Both types of mechanosensitive ion channels exert their effects through the ions they conduct in response to mechanical force and the downstream signaling pathways in vasculature. 

Clinical trials for drugs specifically targeting these mechanosensitive ion channels are being run in the cardiovascular system, as shown in Table 2.

## 4. Cross-Talk between Mechanosensitive Ion Channels and Ca^2+^ Regulatory Proteins in Cardiovascular System

There are many similarities between mechanosensitive ion channels and Ca^2+^ regulatory proteins in the regulation of intracellular Ca^2+^. To date, many scientific reports have shown that they both can cross-talk to each other so that intracellular Ca^2+^ signaling can be precisely coordinated in cardiovascular health and diseases (as listed in Table 3). For some physiological activities, both types of proteins can even physically interact with each other to regulate Ca^2+^ concentration. 

### 4.1. Piezo and SERCA 

Pizeo and SERCA have been found to cross-talk to each other through physical interaction in endothelial cells. SERCA2 inhibits Piezo1-dependent endothelial cell migration through a 14-residue linker region [18]. Mutations in this linker impair the interaction between Piezo1 and SERCA2. As a consequence, the mechanical stimulation of Piezo1 channel activity is significantly weakened. In addition, a synthesized linker peptide also disrupts the regulatory role of SERCA2 in Piezo1 channel, providing further evidence that the linker mediates the physical interaction between SERCA2 and Piezo1 [18]. In Piezo1-deficient elegans, the interference of ER Ca^2+^ stores by SERCA RNAi results in severe reproductive defects [151], reiterating that the importance of cross-talk between Piezo1 and SERCA in the maintenance of physiological functions. When overexpressed human Piezo1 were stimulated by shear stress with or without the presence of SERCA inhibitor thapsigargin in HEK293 cells, Ca^2+^ measurement experiments showed that nearly 72% of the increase in Ca^2+^ is from the mechanically activated Piezo1 while 28% comes from the Ca^2+^ store depletion due to SERCA inactivation [152], suggesting that SERCA inactivation can upregulate Piezo1 activity. Our unpublished data in pulmonary artery SMCs indicates that the substitution of the SERCA2 C674 reactive thiol by serine increases the activity of Piezo as well. This further supports the regulatory role of SERCA2 in mechanically-activated Piezo1 activity, though the exact molecular mechanism remains to be investigated. So far, the interactions between Piezo and SERCA have not been sufficiently reported in the development of cardiovascular disease. Considering the overlapping roles of Piezo and SERCA at the lesion site in some cardiovascular disease such as arterial hypertension and aneurysms, we postulate that both proteins also cross-talk to regulate Ca^2+^ signaling, albeit rather differently, in these diseases.

### 4.2. TRP and NCX

NCX is involved in the regulation of intracellular Ca^2+^ by triggering the Ca^2+^ entry mediated by TRP-formed SOC channels. Pulmonary hypertension, as an example, is closely related to both NCX and TRP proteins. Dysfunction of either protein disrupts the Ca^2+^ homeostasis, which changes contraction, proliferation, and migration in SMCs of pulmonary artery. Gradually the hypertension is developed [136,137]. 

The cross-talk between TRP channels and NCX can regulate contractile dysfunction and spontaneous ectopic [119]. Growing evidence indicates that NCX cannot function properly without the involvement of TRP channels. TRP channels directly interact with NCX to regulate the Ca^2+^ homeostasis. Co-immunoprecipitation experiments show the co-localization of TRPC3 and NCX1, suggesting that TRPC3 cross-talks to NCX1 through physical interaction in the Ca^2+^ regulation [119]. Glutathione s-transferase pull-down assays consistently replicate the natural TRPC3/NCX1 complex in cardiomyocytes. The NCX-mediated Ca^2+^ signaling is significantly reduced in cells with negative TRPC3 expression [153]. In addition to physical interactions, there is also functional coupling underlying the cross-talk between these two types of proteins. Mechanical stimulation activates TRPV1, TRPV2 or TRPV4 and then Ca^2+^ entry is induced. The increased intracellular Ca^2+^ concentration in turn activates NCX to extrude the cytoplasmic Ca^2+^ [154]. NCX and TRP can also be synergistically involved in the Ca^2+^ entry by cooperatively mediating the hypoxia-induced increase in intracellular Ca^2+^ and then causing vasoconstriction [137]. However, the NCX inhibitor KB-R7943 can only be effective in inhibiting TRPC3, TRPC5 or TRPC6. Also, the coupling of TRPM4 with the reverse mode of NCX can induce atrial arrhythmia [138]. However, it should be noted that not all types of TRP channels are functionally coupled to NCX [155]. 

### 4.3. TRP and Orai

TRP and Orai are both located in the cell membrane [139]. The interaction between TRP and Orai is often inseparable from STIM1. A summary of TRP, Orai1 and STIM revealed that these three proteins can also interact with each other or together. STIM can activate both Orai1 and TRP, and then either TRPC1-STIM1 or Orai1-STIM1-TRPC complex is formed to mediate SOCE [139]. Some experimental evidence have suggested that the function of TRPC1 is not only dependent on STIM1, but also Orai. The knockdown of Orai protein disrupts the normal function of TRPC channels [36]. As SOCE has a profound effect on the development of pulmonary hypertension, inhibition of any such protein can be used as an effective treatment of pulmonary hypertension [140]. In addition, SOCE is also involved in thrombosis thrombin production [141]. 

### 4.4. TRP and STIM

TRP can cross-talk to STIM1 through physical interaction between them [139]. The interaction between TRP and STIM is often triggered by intracellular Ca^2+^ change. Upon depletion of Ca^2+^ stores in the ER, STIM1 can form a dimer and then activate TRPC channels. However, the TRPC channel function is not always dependent on STIM1 and therefore the interaction between STIM1 and TRPC may be absent in some cells [36]. When they form a complex together, they can coordinate Ca^2+^ signal to regulate another protein. It has been observed that TRPC1, TRPC2, or TRPC4 can interact directly with the ezrin/radixin/moesin domain of STIM1 [156]. Interaction between TRPC1 protein and STIM1 could stimulate phospholipase C and then induce TRPC1 channel activation in vascular SMCs [157]. Fluorescence resonance energy transfer (FRET), immunoprecipitation and total internal reflection fluorescent microscope (TIRFM) experiments have been extensively carried out to demonstrate that TRPC1 strongly co-localizes and interacts with STIM1 after depletion of ER Ca^2+^ stores. In addition, the Orai1-mediated Ca^2+^ entry into the cytosol can also trigger TRPC1 interaction with STIM1. In this case, each TRPC1 tetramer binds to two STIM1s and the ^639^DD^640^ of TRPC1 C-terminus is then directly interacted by the ^684^KK^689^ region of STIM1 C-terminus [142]. Caveolin-1 as a key scaffold in the plasma membrane where TRP proteins reside has been reported to underlie the formation of TRPC-STIM1 complex. Its knockdown prevents TRPC binding to STIM1 [158]. Though the interaction of TRPC1 with STIM1 may be required for SOCE activation, the knockdown of TRPC1 decreases SOCE activity by approximately 60%, while the deletion of STIM1 completely abolished SOCE activity [142]. The interaction between TRP and STIM is not always dependent on Ca^2+^ store depletion. When the EF-hand of STIM1 is mutated, it spontaneously aggregates with TRPC1 and then activates TRPC1 [159]. The interaction between TRP and STIM is implicated in the progression of many diseases, particularly some cardiovascular diseases such as arterial hypertension, atherosclerosis, or thrombosis [141,143,144,145,146].

### 4.5. TRP and IP3R

The IP3R-initiated Ca^2+^ depletion is followed by activation of TRP channels to promote Ca^2+^ influx, which supports the notion that IP3R cross-talks to TRP channels to regulate intracellular Ca^2+^ [67]. This is further confirmed by the interaction of IP3R with TRP [71]. The interaction between TRP and IP3R has been studied in more details with structural approaches. It has been found that TRPV4 or other member of TRP family can interact with IP3Rs. The IP3R interacts with TRP protein in a Ca^2+^-dependent manner. Also, IP3R has differential affinity to various TRP member. This affinity ranges from 10 nm for TRP2 to 290 nm for TRP6 [160]. Prior studies showed that the interaction between IP3R and TRP could not be achieved without Homer1. Homer1 is a scaffolding protein that enhances the physical interaction between TRPC1 and IP3R [161]. Co-immunoprecipitation and co-localization both demonstrate the direct interactions between Homer1b/c isoforms, IP3R and TRPC2 [162]. The coupling of IP3R to TRP forms the mechanism underlying the physiological vasoconstriction or the endothelin-1-induced hypertensive vasoconstriction [147,148]. Therefore, such coupling and cross-talk not only contribute to vascular SMCs contraction, but also is associated with hypertensive resistance [163,164].

### 4.6. TRP and SERCA

Though the direct physical interaction between TRP channels and SERCA has not yet reported, numerous studies have shown that they both can cross-talk to each other. SERCA pump inhibitor thapsigargin blocks the activation of TRPC7 channel by diacylglycerol or the activation of TRPC3 channel [165]. The SERCA modulator 2-aminoethyl diphenylborinate also has a significant inhibitory effect on TRPV6 activity [166]. The Ca^2+^ signaling compensatory mechanism in which the transcription of TRPC4 and TRPC5 are increased after SERCA silencing has been proposed to be responsible for cardiac development and hypertrophy [150]. A similar compensatory mechanism also occurs under hypoxia. Hypoxia inhibits SERCA activity and increases the Ca^2+^ influx through TRPC6 [167]. The expression of TRPC1 is inversely correlated with SERCA2 [168]. SERCA also regulates the expression and activity of TRP channels. Silencing SERCA2 increases the transcription and activity of TRPC4 and TRPC5 in cardiomyocytes, suggesting that intracellular Ca^2+^ can be compensated by TRP channels when the Ca^2+^ in the store is insufficient [150]. However, it remains unclear whether down-regulation of TRPC4/5 inversely up-regulates the expression of SERCA2. The cross-talk between the TRP channel and SERCA contributes to the development and function of heart. It has been evidenced that the rapid cyclic change of intracellular Ca^2+^ concentration during cardiac pulsation is a consequential result of temporal cross-talk between SERCA and TRP. Thus these proteins could be promising targets in treating cardiac disease [149].

## 5. Conclusions

The multifaceted effects of the Ca^2+^ signaling pathway in both physiology and pathology require the dynamic cross-talk between mechanosensitive ion channels and Ca^2+^ regulatory proteins. Ca^2+^ regulatory proteins are diverse and widely distributed in the cells, mostly on the cell membrane or in ER. Notably, certain pathogenic factors are largely activated by changes in intracellular Ca^2+^ concentrations, and pharmacological modulation of these proteins has emerged as an attractive strategy to prevent cellular dysfunction and tissue remodeling, and eventually to treat cardiovascular disease. Mechanosensitive ion channels play a dominant role in endothelium-dependent vascular development and blood pressure regulation, vascular tension control, angiogenesis and the atherosclerotic process. When either mechanosensitive ion channel or Ca^2+^ regulatory protein is activated, a Ca^2+^-related pathway is often triggered and then the cross-talk between both proteins is frequently initiated. In addition, it is interesting to observe that in some cardiovascular diseases, such as hypertension, atherosclerosis or aneurysms, mechanical stress or shear stress is altered, and inherent activation of the mechanosensitive ion channels are reported. Accordingly, Ca^2+^ regulatory proteins are communicated by the channels on a temporal and spatial basis. In some cells, when mechanosensitive ion channel expression is defective or disrupted, the inability to sense the mechanical stimuli directly affects the regulation of downstream Ca^2+^-related pathways, which leads to defects in some physiological functions. The cross-talk between these mechanosensitive ion channels and Ca^2+^ regulatory proteins in the cardiovascular system is therefore not only relevant to the maintenance of health but is also involved in the development of diseases.

The summary of recent articles on the role of mechanosensitive ion channels and Ca^2+^ regulatory proteins in cardiovascular health and disease through the regulation of Ca^2+^ related pathways reveals an apparent cross-talk between both types of proteins. The cross-talk occurs with either physical interaction or functional coupling. Especially, the physical interaction in such cross-talk has emerged to be important for the cardiovascular physiology or pathology. We have summarized some interactions between mechanosensitive ion channels and Ca^2+^ regulatory proteins. The study of protein–protein interaction depends on FRET, TIRFM, and immunoprecipitation techniques. With the comprehensive application of protein-protein interaction methods and the development of electron microscopy technology, we expect that more interactions between mechanosensitive ion channels and Ca^2+^ regulatory proteins will be discovered. In addition, the functional coupling in the cross-talk between the channels and Ca^2+^ regulatory proteins will also be unraveled with functional assays combined with appropriate animal models. These studies will certainly be useful in identifying novel therapeutic approaches for treatment of cardiovascular diseases.

## Figures and Tables

**Table 1:** Current clinical trials for drugs targeting these Ca^2+^ regulatory proteins in the cardiovascular system according to the ClinicalTrials.gov website (available online and accessed on 12 August 2021).

Ca^2+^ Regulatory Proteins	Treatment	Cardiovascular Disease	Phase
SERCA	AAV1/SERCA2a (MYDICAR) [92]	Ischemic cardiomyopathy; non-ischemic cardiomyopathy; heart failure; cardiomyopathies	Phase 2
	MYDICAR-single intracoronary infusion [93]	Heart failure, congestive; ischemic cardiomyopathy; non-ischemic cardiomyopathy	Phase 2
	MYDICAR [94]	Chronic heart failure	Phase 2
	SRD-001 [95]	Congestive and systolic heart failure	Phase 1/Phase 2
	Istaroxime [96]	Heart failure [97]	Early phase 1
NCX	MYDICAR [98]	Chronic heart failure	Phase 2
Orai	No resource	No resource	No resource
STIM	No resource	No resource	No resource
IP3R	No resource	No resource	No resource

**Table 2:** Current clinical trials for drug specifically targeting these mechanosensitive ion channels are run in the cardiovascular system, according to the ClinicalTrials.gov website (available online and accessed on 12 August 2021).

Mechanosensitive Ion Channels	Treatment	Cardiovascular Disease	Phase
Piezo	Senicapoc (synonyms: ICA-17043; 2,2-bis-(4-fluorophenyl)-2-phenylacetamide) [129]	Dehydrated hereditary stomatocytosis (related to pulmonary hypertension [130])	Phase 1/Phase 2
TRPV1	Capsaicin and mechanical stimulation with Von Frey filaments [131]	Cystinosis (related to portal hypertension [132])	Early phase 1
TRPV4	GSK2798745 [133]	Heart failure	Phase 2
TRPM8	Menthol [134]	Hypertension [135]; Prehypertension	Phase 2/Phase 3

**Table 3:** Summary of the cross-talk between mechanosensitive ion channels and Ca^2+^ regulatory proteins in cardiovascular health and disease.

Mechanosensitive Ion Channels	Ca^2+^ Regulatory Proteins	Cardiovascular Health	Cardiovascular Disease
Piezo	SERCA	Inhibition of piezo-dependent endothelial cell migration [18]	No resource
TRP	NCX	Cell contraction, proliferation and migration [136]; vasoconstriction [119,137]	Pulmonary hypertension [136,137]; arrhythmia [138]
	Orai	Participation in SOCE [139,140]	Pulmonary hypertension [140]; thrombosis [141]
	STIM	SOCE activation [142]	Hypertension and atherosclerosis [143,144,145]; thrombosis [141,146]
	IP3R	Vasoconstriction; regulating the VGCC function [147,148]	Hypertension [148]
	SERCA	Heartbeat and heart development [149]	Cardiac hypertrophy [150]
